# Assessment of Concordance and Discordance Among Clinical Studies Posted as Preprints and Subsequently Published in High-Impact Journals

**DOI:** 10.1001/jamanetworkopen.2021.2110

**Published:** 2021-03-18

**Authors:** Xiaoting Shi, Joseph S. Ross, Nishita Amancharla, Joshua D. Niforatos, Harlan M. Krumholz, Joshua D. Wallach

**Affiliations:** 1 Department of Environmental Health Sciences, Yale School of Public Health, New Haven, Connecticut; 2 Section of General Internal Medicine, Yale School of Medicine, New Haven, Connecticut; 3 Yale College, New Haven, Connecticut; 4 Department of Emergency Medicine, The Johns Hopkins University School of Medicine, Baltimore, Maryland; 5 Section of Cardiovascular Medicine, Yale School of Medicine, New Haven, Connecticut

## Abstract

This cross-sectional study examines the concordance between clinical studies posted as preprints and subsequently published in high-impact journals, including key study characteristics, reported results, and study interpretations.

## Introduction

Dissemination of clinical and health science research as preprints—that is, preliminary reports of studies that have not yet undergone peer review—has grown rapidly since the launch of the medRxiv preprint server in 2019.^1,2^ Although not all preprints will subsequently be published in peer-reviewed journals, among those that are, the extent of the changes to studies’ reporting of design, conduct, and results remains unknown.^3^ Accordingly, in this cross-sectional study, we examined the concordance between clinical studies posted as preprints and subsequently published in high-impact journals (preprint–journal article pairs), including key study characteristics, reported results, and study interpretations.

## Methods

This study did not require institutional review board approval because it was based on publicly available information, in accordance with 45 CFR §46. Informed consent was not needed because no patient data were used.

We used the bioRxiv/medRxiv Application Programming Interface to identify all preprint–journal article pairs from medRxiv’s inception on June 11, 2019, through August 13, 2020. We limited our sample to clinical trials, prospective and retrospective observational studies, and systematic reviews with meta-analyses reporting health-related outcomes that were published in peer-reviewed journals with a 2019 impact factor greater than 10 according to InCites Journal Citation Reports. For preprint–journal article pairs, we abstracted the number of authors; first and senior authors; funding, conflict of interest, and institutional review board disclosure statements; and sample size (number of patients for trials and observational studies or number of studies for meta-analyses). In addition, we identified the prespecified primary end points, corresponding results from inferential analyses, and overarching abstract-level conclusions and study interpretations; for studies without clearly defined end points, we recorded all abstract-level end points and results or categorized the study as purely descriptive. Building on previously published methods,^4^ disclosure statements, sample size, number of authors, and end points were considered concordant if they contained the same information or had numerical equivalence. Results from inferential analyses were considered discordant if effect estimates and/or 95% CIs or *P* values changed. For preprint–journal article pairs with descriptive results, we did not determine whether results were concordant or discordant. Descriptive analyses were conducted using Excel software version 16.0 (Microsoft) from November 2020 to January 2021.

## Results

Among 8941 preprints posted on medRxiv, 944 had been subsequently published in a peer-reviewed journal as of our search date. Of these, 47 were clinical studies published in a high-impact journal (Table 1). Most preprint–journal article pairs were retrospective studies (35 of 47 pairs [74%]) and were related to the coronavirus disease 2019 pandemic (28 of 47 pairs [60%]).

**Table 1.** Characteristics of Clinical Study Preprints on medRxiv With a Corresponding Peer-Reviewed Journal Article in a High-Impact Journal^a^

Characteristics	Studies, No. (%) (N = 47)
Clinical study design	
Retrospective studies	35 (74)
Prospective studies	10 (21)
Systematic reviews with meta-analyses	2 (4)
Related to coronavirus disease 2019	
Yes	28 (60)
No	19 (40)
medRxiv subject area	
Infectious diseases (except HIV/AIDS)	11 (23)
Epidemiology	8 (17)
Cardiovascular medicine	5 (11)
Neurology	4 (9)
Genetic and genomic medicine	4 (9)
Rheumatology	3 (6)
Oncology	3 (6)
Intensive care and critical care medicine	3 (6)
Respiratory medicine	2 (4)
Others^b^	4 (9)
Dissemination characteristics^c^	
Preprint versions, median (range), No.^d^	1 (1-4)
Preprint comments, median (range), No.	0 (0-13)
Preprint usage, median (IQR), No.	
Overall views	4022 (1311-15 218)
Overall downloads	1379 (501-5794)
Altmetric score, median (IQR)	
Preprints	26 (4-144)
Journal articles	63 (24-366)
Time from the first preprint version to publication, median (IQR), d	52 (33-136)

Abbreviation: IQR, interquartile range.

^a^ High impact refers to an InCites Journal Citation Reports 2019 impact factor greater than 10. Thirty-one unique high-impact journals were identified. The most common journal was *The American Journal of Human Genetics* (4 articles).

^b^ Includes hematology, neurology, pathology, and pediatrics.

^c^ Data were updated December 7, 2020.

^d^ For preprints with multiple versions posted on medRxiv, we used the most recent version of preprint prior to journal acceptance. Preprints were excluded if they were only posted after the date of journal acceptance.

Among the 47 preprint–journal article pairs, 46 (98%) reported the same first and senior authors and 36 (77%) had the same total number of authors (Table 2). Most of the pairs had concordant funding statements (39 pairs [83%]) and institutional review board disclosures (44 pairs [94%]). Of the 17 pairs (36%) with discordant conflict of interest statements, there were 8 where only the journal articles contained a conflict of interest disclosure. Among the 44 pairs reporting sample sizes in both sources, 38 were concordant. Sample sizes were larger in the journal articles for nearly all discordant pairs (5 of 6 pairs [83%]).

**Table 2.** Concordance Between Clinical Study Preprints and Articles Published in High-Impact Journals

Characteristic	Preprint–journal article pairs, No. (%) (N = 47)
Authors	
First and senior author	
Concordant	46 (98)
Discordant	1 (2)
No. of authors	
Concordant	36 (77)
Discordant	11 (23)
More authors in the preprint	3 (27)
More authors in the journal article	8 (73)
Funding statements	
Concordant	39 (83)
Identical funding statements in the preprint-journal article pairs	34 (87)
No funding statements in the preprint-journal article pairs	5 (13)
Discordant	8 (17)
Funding statement in the preprint only	0 (0)
Funding statement in the journal article only	1 (13)
More funders disclosed in preprint	1 (13)
More funders disclosed in journal article	3 (38)
Other^a^	3 (38)
COI statements	
Concordant	30 (64)
Identical COI statements in the preprint-journal article pairs	30 (100)
No COI statements in the preprint-journal article pairs	0 (0)
Discordant	17 (36)
COI statement in the preprint only	0 (0)
COI statement in the journal article only	8 (47)
More COIs disclosed in preprint	2 (12)
More COIs disclosed in journal article	5 (29)
Other^a^	2 (12)
IRB statements	
Concordant	44 (94)
Identical IRB statements in the preprint-journal article pairs	37 (84)
No IRB statements in the preprint-journal article pairs	7 (16)
Discordant	3 (6)
IRB statement in the preprint only	1 (33)
IRB statement in the journal article only	1 (33)
More IRB statements in journal article	1 (33)
Sample size	
Could not be compared^b^	3 (6)
Concordant	38 (81)
Discordant^c^	6 (13)
Larger sample size in the preprint	1 (17)
Larger sample size in the journal article	5 (83)
Primary end points	
Concordant	44 (94)
Discordant	3 (6)
Results for primary end points	
Could not be compared^b^	10 (21)
Concordant	25 (53)
Discordant	12 (26)
Effect estimates discordant; direction of effect and statistical significance concordant	7 (58)
Effect estimates discordant; direction of effect and/or statistical significance discordant	2 (17)
No. of outcomes or No. of reported outcomes discordant	3 (25)
Study interpretation	
Could not be compared	1 (2)
Concordant	45 (96)
Discordant	1 (2)

Abbreviations: COI, conflict of interest; IRB, institutional review board.

^a^ Other refers to preprint–journal article pairs where more information is disclosed for certain authors in the preprint (or journal article) and less information is disclosed for other authors in the preprint (or journal article). When evaluating funding and COI disclosures, we only compared authors who were concordant between preprints and journal articles.

^b^ Sample size, results for primary end points, and study interpretations were classified as could not be compared when preprint–journal article pairs contained unclear information or reported descriptive results (ie, nonnumerical or noninferential analyses).

^c^ Four preprint–journal article pairs had ascertainment time changes.

Overall, 44 preprint–journal article pairs (94%) reported concordant primary end points (Table 2). Ten of 47 pairs (21%) were descriptive (noninferential) studies without clearly comparable numerical results. Among the remaining 37 pairs reporting primary results from inferential analyses, 12 (32%) reported discordant results; 7 had effect estimates that were in the same direction and were statistically consistent. Nearly all pairs (45 of 46 pairs [98%]) with clearly stated study interpretations were concordant, including all pairs with discordant results.

## Discussion

Although preprints are preliminary reports that should be critically assessed, similar to prior studies of text content,^5^ our findings suggest that the overarching study interpretations, as well as other study design details described in preprints of clinical studies that are subsequently published in high-impact journals, did not change markedly. Our study was limited to medRxiv preprints published in high-impact journals and may represent the best-case scenario; we did not account for preprints never submitted to or rejected by journals, nor could we determine whether changes improved research reporting accuracy. Given concerns about the role of preprints in clinical research,^3,6^ future evaluations should monitor the concordance of preprint–journal article pairs of clinical studies.
